# Awake Prone Decubitus Positioning in COVID-19 Patients: A Systematic Review and MetaAnalysis

**DOI:** 10.2478/jccm-2023-0014

**Published:** 2023-05-08

**Authors:** Agustin García, Rita Galeiras, Sonia Pertega-Díaz

**Affiliations:** 1 A Coruna University Hospital, A Coruña, Spain; 2 University of A Coruna, A Coruña, Spain

**Keywords:** awake, prone position, COVID-19, mortality, mechanical ventilation

## Abstract

To date, recommendations for the implementation of awake prone positioning in patients with hypoxia secondary to SARSCoV2 infection have been extrapolated from prior studies on respiratory distress. Thus, we carried out a systematic review and metaanalysis to evaluate the benefits of pronation on the oxygenation, need for endotracheal intubation (ETI), and mortality of this group of patients. We carried out a systematic search in the PubMed and Embase databases between June 2020 and November 2021. A randomeffects metaanalysis was performed to evaluate the impact of pronation on the ETI and mortality rates. A total of 213 articles were identified, 15 of which were finally included in this review. A significant decrease in the mortality rate was observed in the group of pronated patients (relative risk [RR] = 0.69; 95% confidence interval [CI]: 0.480.99; p = 0.044), but no significant effect was observed on the need for ETI (RR = 0.79; 95% CI: 0.631.00; p = 0.051). However, a subgroup analysis of randomized clinical trials (RCTs) did reveal a significant decrease in the need for this intervention (RR = 0.83; 95% CI: 0.710.97). Prone positioning was found to significantly reduce mortality, also diminishing the need for ETI, although this effect was statistically significant only in the subgroup analysis of RCTs. Patients’ response to awake prone positioning could be greater when this procedure is implemented early and in combination with noninvasive mechanical ventilation (NIMV) or highflow nasal cannula (HFNC) therapy.

## INTRODUCTION

Prone positioning is a validated strategy in the treatment of patients with acute respiratory distress syndrome (ARDS) and recommended in critically ill, sedated patients on invasive mechanical ventilation (IMV). There are several mechanisms that may contribute to the benefit of this positioning, including a more homogeneous transpulmonary pressure distribution, an improved ventilation/perfusion ratio due to the decreased shunt, pulmonary recruitment secondary to the reduced compressive weight, and an improved right ventricular function. Considering the above pathophysiological reasoning, some authors have proposed the use of this strategy in non-intubated patients with hypoxemic respiratory failure. In this regard, as a result of the high number of patients presenting with lung damage caused by the severe acute respiratory syndrome coronavirus 2 (SARSCoV2) since the beginning of the pandemic, a large number of studies have been conducted with the aim of analyzing the benefits and tolerance of this maneuver in awake patients requiring oxygen supplementation.

The findings of most of these studies including patients with the 2019 coronavirus disease (COVID19) suggest that the prone positioning had positive effects on oxygenation levels. However, although reviews have already been published in this respect [1–5], uncertainties remain as to whether these changes persist following resupination, as well as concerning their impact on mortality or the prevention of respiratory support escalation. Awake prone positioning could be included as part of a package of therapeutic measures, particularly when combined with noninvasive mechanical ventilation (NIMV) and highflow nasal cannula (HFNC) therapy. However, there is no evidence as to what the most appropriate protocol to follow might be considering the patients’ tolerance or which patients are the best candidates for this strategy.

Thus, the aim of this review is to gather clinical evidence available thus far on the benefits of awake prone positioning in COVID19 patients with hypoxia based on their oxygenation improvements, need for IMV, and inhospital mortality.

## MATERIALS AND METHODS

We carried out a systematic review and metaanalysis of the scientific literature to gather the available evidence thus far on the usefulness of awake prone decubitus positioning in adult patients with hypoxemic respiratory failure secondary to a SARSCoV2 infection.

We used the Preferred Reporting Items for Systematic Reviews and MetaAnalyses (PRISMA)[6] criteria during the conduct of this review and metaanalysis.

### Search Strategy

We performed a comprehensive, systematic search of medical literature published between January 2020 and November 2021 to identify studies exploring the usefulness of prone decubitus positioning in nonintubated patients hospitalized for COVID19.

The literature search was carried out in the Excerpta Medica Database (EMBASE) and PubMed, using the following search keywords: “awake”, “prone position”, “prone positioning”, and “COVID-19”. Our search strategy was supervised by the local medical librarian.

### Study Selection

Studies were deemed relevant for this review if they met the following inclusion criteria: studies including patients aged ≥18 years who had been admitted to the hospital with respiratory failure due to COVID-19, were awake, did not require invasive ventilation, and in whom the benefit of the prone positioning strategy had been studied.

The following exclusion criteria were considered:

— Studies including less than 50 patients.
— Systematic reviews, metaanalyses, case series, recommendations or guidelines, conferences or protocols, reviews, or commentaries.
— Studies focusing on an objective other than analyzing the efficacy of awake prone positioning in COVID-19 patients.
— Studies published in a language other than Spanish or English.

**Fig. 1.**
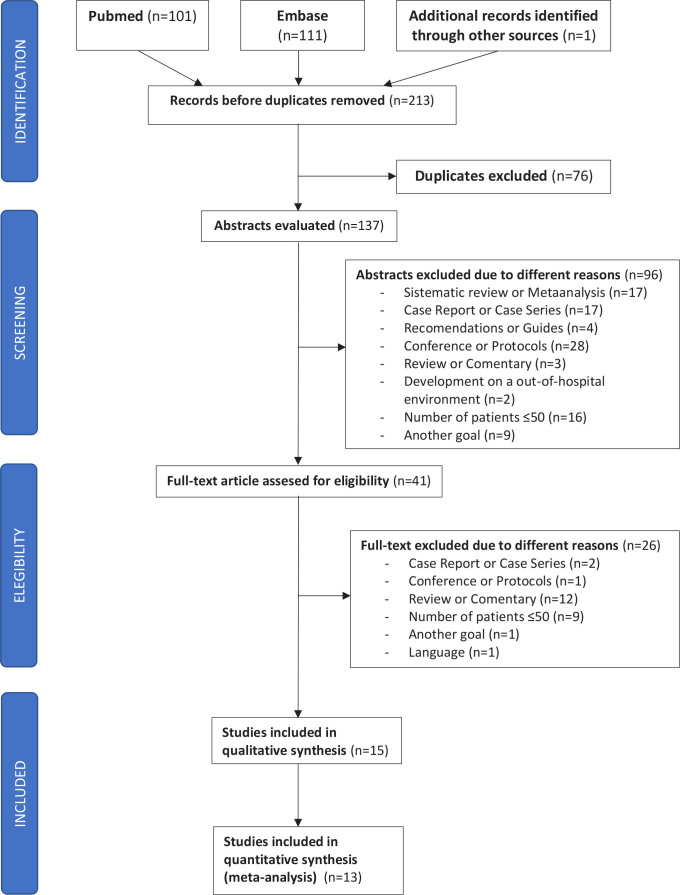
PRISMA Flow Diagram of the Study Select

Two reviewers independently carried out the study selection process considering the above criteria. The titles of the articles (212 references) were first reviewed, with 76 duplicate articles being excluded from our analysis. The abstracts of the remaining articles (137 references) were then reviewed, with 96 articles being excluded for varied reasons described in **Figure 1**.

The next step was to analyze the full text of each article that seemed to be eligible according to the first selection phase (41 articles), with a total of 26 being excluded after this process. Disagreements regarding the selected studies were resolved by consensus between the two reviewers.

One article found by an external search was also added, with a final total of 15 articles being included in the review (**Supplementary Material Table 1**).

### Data Collection

We extracted the data from each eligible study and used predesigned data collection forms to manage them.

The information extracted from the selected studies, whenever available, included the following data: the first author, the year and country of publication, the study design, the patient inclusion criteria used for each study, the sample size, the total number of patients placed in a prone position in each study, the type of healthcare setting, associated comorbidities, the oxygen supplementation interface used, the prone positioning protocol applied, the rate of endotracheal intubation (ETI), the mortality rate, as well as the oxygenation and ventilation parameters available.

### Quality Assessment of the Studies

A blinded and independent quality assessment of all studies included in the review was performed by two of the researchers (RGV and AGD). Discrepancies in this assessment were resolved by consensus. The Jadad scale [7] was used in the case of randomized clinical trials (RCTs) and the NewcastleOttawa scale [8] was used for observational studies. The Jadad scale evaluates the methodological quality of a clinical trial through five items assessing aspects related to biases, including the randomization, the blinding of the patients and researcher to the treatment, and the description of patients lost to followup. A clinical trial is considered to be of low quality if it scores less than 3 points in this scale. On the other hand, the NewcastleOttawa scale consists of eight items divided into three dimensions (comparison, selection, and outcomes). According to this scale, the risk of bias can be rated as high (13 points), moderate (46 points), or low (79 points), with studies with a score equal to or greater than 7 considered to be of high quality.

### Data Analysis

The statistical analysis was carried out using package *meta* of software R (version 2.15.1). A *p* value <0.05 was considered to be statistically significant.

A metaanalysis of the studies included in the review was performed applying the randomeffects model described by DerSimonian and Laird [9] with the aim of considering the heterogeneity among such studies. As summary measures of effect, we considered the cumulative incidence of ETI and mortality, as well as the relative risk (RR) of the presence of both events in relation to pronation, together with their 95% confidence interval (CI). DerSimonian and Laird’s Qtest, in addition to the I^2^ index, were used to analyze the heterogeneity among the studies included in the review. I^2^ values of 0%-25%, 26-75%, and 76%-100% were considered indicative of low, moderate, and substantial heterogeneity, respectively.

To evaluate potential sources of inter-study heterogeneity, additional random effects models were developed after stratifying the data by study design (i.e., RCTs vs. prospective or retrospective observational studies) and geographical region.

Publication bias was assessed using a funnel scatter plot, as well as Egger’s and Begg’s tests [10–11].

We also performed a sensitivity analysis to determine the influence of each individual study on the overall result, as well as a subgroup analysis according to the study design.

## RESULTS

After applying the selection process described above, 15 studies with a pooled population of 3912 patients were included in the qualitative analysis, and 13 studies with a total sample size of 3090 patients were included in the quantitative analysis. All of these studies had been published between June 2020 and November 2021, and their main characteristics are outlined in **Table 1**. Five were carried out in Europe [12–16], another five in the United States of America (USA) [17–21], three in South America [22–24], one in Asia [25] and one was multicontinental [26]. Most were multi-center studies (n = 9) [12–14;17–18;21;23;25–26], five were singlecenter [15;19–20;22;24], and one bicenter [16]. As for their design, most were retrospective cohort studies (n = 8) [12;16;18–20;22–24] followed in frequency by RCTs (n = 4) [13;17;25–26] prospective cohort studies (n = 2) [14–15] and, finally, a retrospective casecontrol study (n = 1) [21]. Nine of these studies included a control group.

**Table 1.** Characteristics of the Studies and Patients included.

Reference ID, study, and year	Country	Study design	Rating scales (NOS for observational studies and Jadad scale for RCTs)	Total N	Pronated N/nonpronated N	Setting	Age	Male n (%)	Mean BMI (kg/m^2^) ± SD or median (IQR) or n (%)	Median APACHE II score (IQR) or mean ±SD	Medium SOFA score (IQR)	Oxygen therapy interface
1 DueñasCastell et al., 2021 (42)	Colombia	Retrospective cohort study	8	212	212	GW, ER, or ICU	63 (median) IQR: 48.873	142 (67%)	BMI >30: 11/212 (n/N) (5.2%)		Overall: 4 (35.25)	NC or VMK with a reservoir
2 Kaur et al., 2021 (37)	USA	RCT	3	125	125 Premature pronation: 92 Delayed pronation: 33		62 (mean) ± 11.9 (SD)	79 (63.2%)	30 (mean) ± 5 (SD)		Overall: 3 (24.5)	HFNC
3 Ehrmann et al., 2021 (46)	Canada, France, Ireland, Mexico, USA, and Spain	RCT	3	1121	564/557	GW, ER, IMCU, or ICU	61.1 (mean)	746 (66.5%)	29.7 (mean) ±4.6 (SD) Int.: 29.7 ±4.6 Con.: 29.7 ±4.6			HFNC or NIMV
4 PerezNieto et al., 2021 (43)	Mexico and Ecuador	Retrospective cohort study	7	827	505/322	GW, ER, or ICU	54.3 (mean) ± 14.2 (SD)	600 (72.6%)	BMI >30: 119 (14.4%) Int.: BMI >30: 74 (14.7%) Con.: BMI >30: 45 (14%)			NC, HFNC, or VMK with reservoir
5 Tonelli et al., 2021 (32)	Italy	Retrospective cohort study	7	114	38/76	Respiratory ICU	67 (median) IQR: 3280	80 (70%)	27.5 (median) IQR: 1937 Int.: 26 (1936) Con.: 28 (2037)	Overall: 10 (422) Int.: 11 (422) Con.: 10 (420)	Overall: 4 (27) Int.: 4 (27) Con.: 4 (26)	NIMV, HFNC, and CPAP
6 Jayakumar et al., 2021 (45)	India	RCT	3	60	30/30	ICU	56.05 (mean)	50 (83.3%)	27 (mean) Int.: 28.2 ±5.7 Con.: 25.8 ± 2.6	Int.: 9.5 ±3.6 Con.:8.6±3.1		NC, MV, VMK with reservoir, HFNC, or NIMV
7 Downing et al., 2021 (38)	USA	Retrospective cohort study	8	97	97	GW, ER, or ICU	54 (mean) ± 14 (SD)	60 (61.8%)	31 (mean) BMI <30: 41 (42%) BMI 3040: 37 (38%) BMI >40: 19 (19%)			NC, HFNC, or NIMV
8 Cherian et al., 2021 (39)	USA	Retrospective cohort study	6	59	59	IMCU or ICU	54.5 (median)	39 (66%)	BMI <30: 22 (37.2%) BMI >30: 37 (62.7%)			HFNC or NIMV
9 Rosen et al., 2021 (33)		Sweden	RCT	3	75	36/39	65.48 (median)	55 (73.3%)	28.52 (median)	Int.: 28 (2530) Con.: 29 (2733)		HFNC or NIMV
10 Ferrando et al., 2020 (34)	Spain	Prospective cohort study	8	199	55/144	ICU	62.17 (median)	147 (73.8%)	27.16 (median) Int.: 26.8 (24.831.2)/49 Con.: 27.3 (25.129.4)/120	Int.: 8.5 (613) /46 Con.: 11(814) /107	Int.:4(44)/46 Con.: 4 (45) /116	HFNC
11 Padrao et al., 2020 (44)	Brazil	Retrospective cohort study	7	166	57/109	ER	58.1 (mean) ± 14.1 (SD)	112 (67%)	BMI >30: 89 (54%) Int.: BMI >30: 33 (58%) Con.: BMI >30: 89 (54%)			NC, MV, or VMK with reservoir
12 Coppo et al., 2020 (35)	Italy	Prospective cohort study	7	56	46	GW, ER, or HDU	57.4 (mean) ± 7.4 (SD)	44 (79%)	27.5 (mean) ±3.7 (SD)			CPAP, VM, or VMK with reservoir
13 Jagan et al., 2020 (40)	USA	Retrospective cohort study	7	105	40/65		62.06 (mean)	57 (54.2%)	29.25 (median) Int.: 31.3 (26.437.5) Con.: 28 (24.934.4)	Int.: 7 (49) Con.: 10 (716)	Int.: 2 (23) Con.: 4 (25)	
14 Prud’homme et al., 2021 (36)	France	Retrospective cohort study	8	96	48/48	Outside the ICU	61.5 (mean)	68 (70.8%)	27.5 (mean) Int.: 27 ±5 Con.: 28 ±5			NC or HFNC
15 Nauka et al., 2021 (41)	USA	Retrospective casecontrol study	6	600	164	ICU	61.2 (mean) ± 13.1 (SD)	372 (62%)	29.3 (median) IQR: 25.833.9		Overall: 1 (04)	NC, VMK with reservoir, or HFNC

con., control; CPAP: continuous positive airway pressure; ER, emergency room; GW, general ward; HDU, high dependency unit; HFNC, highflow nasal cannula; ICU, intensive care unit; IMCU, intermediate care unit; int., intervention; NC, nasal cannula; VMK, ventimask; VMK with reservoir, ventimask with reservoir.

**Supplementary Tables 2 and 3** show the results of the quality assessment performed of the RCTs and observational studies included in the systematic review. The overall percentage of agreement between both researchers was 80.0%, with a kappa coefficient of 0.727 (*p* <0.001), indicative of a good degree of agreement. According to the NewcastleOttawa Scale, nine of the 11 observational studies reached the score of 7 points corresponding to highquality studies. As for the RCTs, all of them reached a score of 3 points, indicative of a low risk of bias.

## Characteristics of the Study Populations

The number of patients included in each study ranged from 56 to 1121, with an overall mean of 260.8 patients. The mean age of these patients ranged between 54 and 67 years, and the percentage of male patients ranged between 54.2% and 83.3% (median percentage of 67.0%). With respect to the severity at admission, four studies [12;14;20;25] reported the Acute Physiology and Chronic Health Evaluation II (APACHE II) score of the subjects at admission, and six [12;14;17;20–22] reported their Sequential Organ Failure Assessment (SOFA) score.

The most frequent comorbidities among the pooled population of patients included in the review were obesity (mean prevalence of 55.5%), followed by high blood pressure (HBP) (53.8%), and diabetes mellitus (25.7%) (**Supplementary Table 4**).

The prone positioning protocols applied in the different studies are very heterogeneous and described in **Supplementary Table 5**.

## Effects of Prone Positioning on Oxygenation

Two studies [14;25] provided data on the partial pressure of oxygen/fraction of inspired oxygen ratio (PaO_2_/FiO_2_) before and after pronation of the patients included in both study groups, and three articles [15;18;22] reported this datum before and after applying the pronation maneuver exclusively in the group of pronated subjects. Five papers [17;19;21;23–14] reported data on the peripheral arterial oxygen saturation/fraction of inspired oxygen ratio (SpO_2_/FiO_2_) before and after applying the change of position in the pronated subjects.

In Ehrmann et al.’s metatrial [26], oxygenation indicators improved significantly during the pronation session, with this improvement persisting even after replacing the patient in a supine position. In the study carried out by Coppo et al. [15], this improvement in the oxygenation was maintained in 23 patients (50%; 95% CI: 34.9%65.1%) after resupination; however, the change was not significant compared with the positioning applied prior to the pronation.

In Perez-Nieto et al.’s study [23], the SpO_2_/FiO_2_ was significantly higher after one hour of pronation compared with the baseline values, with a mean difference of 35.03 units (95% CI: 29.9940.06; *p*<0.0001). In their study, Padrao et al. [24] classified 51% of patients as responders according to their SpO_2_/FiO_2_ before and after the pronation. In the study conducted by Kaur et al. [17], the early implementation (within the first 24 hours of starting HFNC supportive therapy) of prone positioning improved both oxygenation and respiratory function, as assessed by the SpO_2_/FiO_2_ and the respiratory rate-oxygenation (ROX) index. Dueñas-Castell et al.[22], on their part, found that the peripheral arterial oxygen saturation (SpO_2_) after applying the pronation maneuver was higher among survivors compared with nonsurvivors (97%; interquartile range [IQR]: 95-99 vs. 91%; IQR: 85-95;*p* <0.01). In contrast, Nauka et al. [21] found no differences in the worst SpO_2_/FiO_2_ before and after pronation in neither the controls nor cases (median difference in the SpO_2_/FiO_2_ of the cases: 3; IQR: −38 vs. median difference in the SpO_2_/FiO_2_ of the controls: 0; IQR: 35). In fact, Jayakumar et al. [25] and Ferrando et al. [14] actually reported a worsening in patient oxygenation following prone positioning (**Table 2**).

**Table 2.** Oxygenation Characteristics

Reference ID, study, and year	Initial PaO_2_/FiO_2_ ratio	Final PaO_2_/FiO_2_ ratio	Initial SpO_2_/FiO_2_ ratio	Final SpO_2_/FiO_2_ ratio	Initial SpO_2_ (%)	Final SpO_2_ (%)	Initial PaO_2_(mmHg)	Final PaO_2_(mmHg)	Initial ROX index (points)	Final ROX index	% of responders
1. DueñasCastell et al., 2021	Survivors: 201.1 Nonsurvivors: 134.1	Survivors: 252.6 Nonsurvivors: 172.4			Overall: 92 (8896) Survivors: 94 (9097) Nonsurvivors: 88 (8092)	Overall: 96 (9298)Survivors: 97 (9599) Nonsurvivors: 91 (8595)			Overall: 5.12 (4.17.25) Survivors: 5.7 (4.7510.2) Nonsurvivors: 4.1 (3.034.6)		
2. Kaur et al., 2021			Premature pronation: 147.98 Delayed pronation: 134.57	Premature pronation: 163.2 (IQR: 132.8211) Delayed pronation: 141.4 (IQR: 105172.5)					Premature pronation: 5.98 Delayed pronation: 4.79	Premature pronation: 7.24 (59.93) Delayed pronation: 5 (3.86.95)	
3. Ehrmann et al., 2021			PP: 147.9 (SD 43.9) SC: 148.6 (SD 43.1)								
4. PerezNieto et al., 2021			Overall: 189.5 (SD 81.6) PP: 182.39 (SD 81.91) SC: 201.1 (SD 89.8)	PP: 217.42 (SD 81.9)							
5. Tonelli et al., 2021	Overall: 149 (78272) PP: 141 (73223) SC: 153 (84232)										
6. Jayakumar et al., 2021	PP: 201.4 ± 118.8 SC: 185.6 ± 126.1	P/F ratio after 2 hours: PP: 198.5 ± 87.6 SC: 171.7 ± 100.6									
7. Downing et al., 2021	Intubated (median): 180 IQR: 96, 238) Not intubated: 286 (IQR 221, 329)	After 24 h (median): Intubated: 109 (IQR 73, 174) Not intubated: 255 (IQR 280, 363)							Intubated: 9.9 (IQR 4, 13.5) Not intubated: 15.9 (IQR 11.5, 20.4	After 24 hours: Intubated: 4.6 (IQR 3.0, 7.9) Not intubated: 15.7 (IQR 8.2, 18.5)	
8. Cherian et al., 2021			Overall: 164.6 Intubated: 100 (95155) Nonintubated: 206 (100293)	Four hours after the pronation: Overall: 157.5 Intubated: 99 (94141) Nonintubated:					Overall: 6.44 Intubated:4 (36) Not intubated: 8 (410)	Overall: 6.44 Intubated: 4 (36) Not intubated: 8 (413)	
9. Rosén et al., 2021	Overall: 115.51 PP: 115.51 (86.25130.5) SC: 115.51 (93.75129.76)		Overall: 154.1 PP: 151 (131174) SC: 157 (136175)		Overall: 93.52 PP 93 (9194) SC: 94 (9295)		Overall: 67.5 PP 66 (57.7572.75) SC: 69 (61.575)				
10. Ferrando et al., 2020	Overall: 114.8 PP: 125 (99187) SC: 111 (83144)	Overall: 95.54 PP: 103 (80125) SC: 92.5 (77125.5)			Overall: 90 PP: 90 (8892) SC: 90 (8894)	Overall: 88.72 PP: 88 (8490) SC: 89 (8692)					
11. Padrao et al., 2020			SpO_2_/FiO_2_, median (P25, P75): PP: 196 (128, 254)	Median (P25, P75): PP: 224 (159, 307)	Median (P25, P75): Overall: 92.5 (90, 94) PP: 92 (88, 93) SC: 93 (91, 95)	Median (P25, P75): PP: 94 (92, 96)			Median (P25, P75): PP: 5.7 (3.9, 7.7)	Median (P25, P75): PP: 7.7 (5.4, 11)	10% increase in the SpO_2_/FiO_2_: same/51 (51%) 10% decrease in the RR: 35/51 (69%) Either: 41/51 (80%)
12. Coppo et al., 2020	SP1 (before pronation): 180.5 (76.6)	**PP1 (pronated):** 285.5 (112.9) **SP2 (resupination)**: 192.9 (100.9)			SP1: 97.2% (2.0)	PP1: 98.2% (2.2) SP2: 97.1%(2.0)	SP1: 117.1 (47.4)	**PP1:** 200.4 (110.9) **SP2:** 121.4 (69.6)			Increase in the PaO_2_/FiO_2_ of SP1 to SP2 23 patients responded to pronation (50%)
14. Prud’homme et al., 2021			Overall: 289 PP: 279+/84 SC: 299 +/45								
15. Nauka et al., 2021			Worse prior to pronation: Cases: 93 (9197) Controls: 98 (95294)	Worse after pronation: Cases: 93 (8996) Controls: 97 (95104)							

PP: group of pronated patients. RR: respiratory rate. SC: group treated with the standard of care.

The variability among the oxygenation indicators analyzed, the differences in the system used to report them in one or both study groups, and the disparities in the patient management timepoints prevented the conduct of a metaanalysis of these data.

## Endotracheal Intubation (ETI)

### Cumulative Incidence of ETI in Pronated Patients

**Fig. 2.**
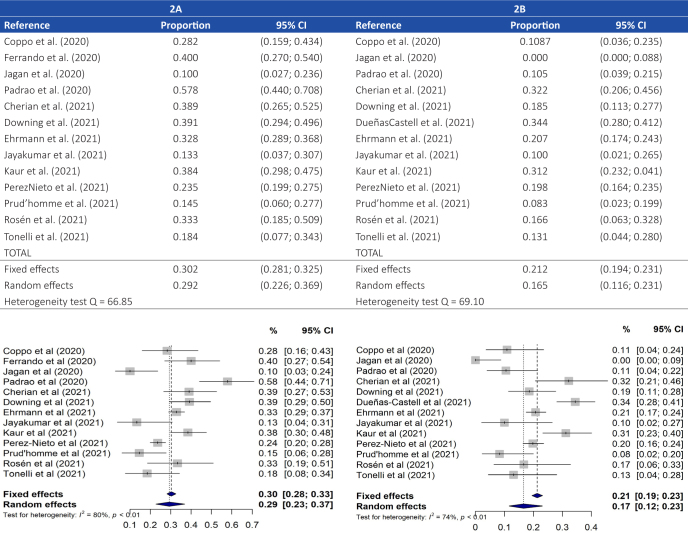
Overall Incidence of ETI (A) and Mortality (B) Among Pronated Patients: MetaAnalysis Results

A total of 13 studies were included in this analysis after excluding two [21–22] from which we failed to obtain the necessary data. Thus, we examined the data of a total of 1700 pronated patients, 515 of whom had been intubated. Our metaanalysis revealed a global rate of ETI of 29.3% (95% CI: 22.6%36.9%), with significant variability among the ETI rates reported by the different authors (10.0%57.8%), as shown in **Figure 2A**. Significant heterogeneity was found across studies (Q-statistic = 60.66; I^2^ = 80.2%; *p* <0.001). A sensitivity analysis revealed no significant variations in the pooled results after excluding or adding each of the analyzed papers (**Supplementary Figure 1**). Although the funnel plot did show some asymmetry caused by the studies with smaller sample sizes (Jayakumar et al. [25], Jagan et al. [20], Prud’homme et al.[16], and Tonelli et al.[12]), the results of Egger’s and Begg’s tests were not statistically significant (*p* = 0.806 and *p* = 0.076, respectively). This suggests the absence of publication bias (**Supplementary Figure 2**).

Since the high heterogeneity observed prevented the conduct of a global meta-analysis, we performed an analysis by subgroups according to the study design. This analysis significantly reduced heterogeneity, revealing a global ETI rate of 32.9% for RCTs, of 34.6% for prospective observational studies, and of 27.2% for retrospective observational studies (*p* = 0.623). However, the heterogeneity was equally remarkable in this last case as shown in **Supplementary Material Figure 3A**. Regarding the geographical region, as shown in **Supplementary Material Figure 3B**, the incidence of ETI ranged between 13.3% in the study performed in Asia and 38.4% in the studies performed in South America, without statistically significant differences (*p* = 0.193). Despite this, heterogeneity among the studies persisted.

## RR of ETI Among Pronated vs. Non-Pronated Patients

**Fig. 3.**
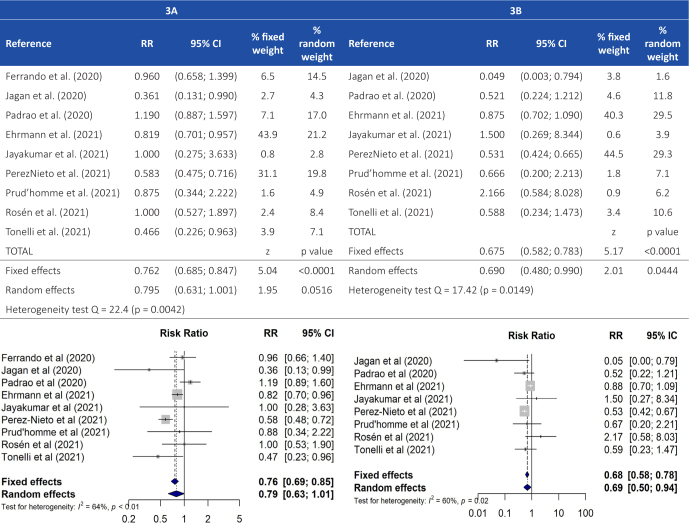
RR of ETI (A) and Mortality (B) Among Pronated vs. NonPronated Patients: MetaAnalysis Results

Nine of the studies (2763 patients) included a control group that allowed to compare the incidence of ETI with that of the group of pronated subjects. Overall, a protective effect of pronation against the need for ETI was observed in most studies, four of them with a statistically significant result, albeit with a very variable effect ranging from a RR of 0.36 to 0.82. Although a similar risk of ETI was found among the controls and subjects of three studies [13–24–25], a non-significant protective effect associated with prone positioning was determined globally (RR = 0.79; 95% CI: 0.631.00) (*p* = 0.051), but with moderate heterogeneity (Q-statistic = 22.08; I^2^ = 63.8%; *p*= 0.005) (**Figure 3A**).

Although the sensitivity analysis showed no significant changes in the overall RR estimation, we found that the studies with the greatest influence were those of Perez-Nieto et al. and Padrao et al.[23–24] When excluding the latter from our analysis, the overall RR was determined to be 0.73 (95% CI: 0.590.91), thus confirming a statistically significant effect of prone positioning and significantly reducing the heterogeneity (I^2^ = 47.2%) (**Supplementary Material Figure 4**). In contrast, our results did not suggest publication bias considering that the values obtained in the Egger’s and Begg’s tests were not statistically significant (*p* = 0.977 and *p* = 0.4655) and no asymmetry was detected in the funnel plot (**Supplementary Material Figure 5**).

In the subgroup analysis (**Supplementary Material Figure 6**), the results yielded by the RCTs demonstrated a statistically significant protective effect of prone positioning against the need for ETI (RR = 0.83; 95% CI: 0.710.97). On the other hand, the only prospective cohort study [14] was found to have the smallest effect of prone positioning against this requirement (RR = 0.96; 95% CI: 0.661.40). Heterogeneity among the retrospective cohort studies was too high to allow for reaching definitive conclusions (I^2^ = 78.5%). In relation to the geographical region, a statistically significant effect was determined in the only study carried out in the USA [20], with a RR of 0.36 (95% CI: 0.130.99), as well as the multicontinental study [26], with a RR of 0.82 (95% CI: 0.700.96). A similar trend was also observed in the studies conducted in European and South American sites, although it failed to reach statistical significance (**Supplementary Material Figure 7**).

## Mortality

### Cumulative Incidence of Mortality Among Pronated Patients

Significantly variable mortality rates, ranging from 0.0% to 34.4%, were identified in the 1857 pronated patients (13 studies) from which mortality data were collected. A total of 395 deaths were reported in all studies, thus resulting in an overall mortality rate in the metaanalysis of 16.6% (95% CI: 11.6%23.1%), as shown in **Figure 2B**. A great heterogeneity was also observed (Q-statistic = 4.93; I^2^ = 74.4%; *p* <0.001). As for the sensitivity analysis, it did not reveal remarkable changes in the pooled result after excluding each of the studies included in the analysis (**Supplementary Material Figure 8**). The funnel plot showed slight asymmetry, even when excluding Jagan et al.’s paper [20], with most studies being located at the left of the plot. In fact, those with a greater sample size were found to report greater mortality rates in the publication bias tests, with Begg’s test yielding statistically significant results (*p* = 0.044) (**Supplementary Material Figure 9**).

Differences between the reported mortality rates were also detected in the subgroup analysis according to the study design, with a 21.4% mean mortality rate in the RCTs (95% CI: 15.2%29.2%), followed by a lower mean rate of 15.2% in the retrospective studies (95% CI: 8.6%25.3%), and of 10.8% in the cohort studies (95% CI: 4.6%23.5%), although these differences were not found to be statistically significant (*p* = 0.225) (**Supplementary Material Figure 10A**). In this case, the heterogeneity continued to be substantial, which limited the validity of the results obtained. In terms of the geographical region, the mean mortality rate was 21.1% (95% CI: 12.3%34.0%) in the South American studies, 11.9% (95% CI: 7.8%17.7%) in the European ones, and 15.3% (95% CI: 4.2%42.6%) in those carried out in the USA. However, these differences were not deemed to be statistically significant either (*p* = 0.082) (**Supplementary Material Figure 10B**).

### RR of Mortality Among Pronated vs. NonPronated Patients

An analysis of the results of the eight studies that reported mortality data for both pronated and control subjects (2564 patients), with 559 pooled deaths, revealed a significant protective effect of this procedure on the mortality rate of the group of pronated patients (RR = 0.69; 95% CI: 0.480.99) (Q-statistic = 17.38; I^2^ = 59.7%; *p* = 0.015) (**Figure 3B**). In general, no major changes were observed in this respect in the sensitivity analysis. The studies with the greatest impact were those carried out by PerezNieto et al. and Ehrmann et al.[23;26], given that exclusion of the first one resulted in an effect of 0.77 (95% CI: 0.511.18) and that of the second one resulted in an effect of 0.62 (95% CI: 0.410.94) (**Supplementary Material Figure 11**). An analysis was also performed to assess the existence of publication bias, with none of the tests yielding statistically significant results (*p*= 0.924 and *p* = 0.901). Furthermore, as no major asymmetries were observed in the funnel plot either, we determined that there is no evidence of publication bias (**Supplementary Material Figure 12**).

After performing a metaanalysis of the results of the retrospective cohort studies, we conclude that prone positioning had a statistically significant protective effect against mortality in their study populations (RR = 0.53; 95% CI: 0.430.65). Despite not reaching statistical significance, this trend was similar for the RCTs. The difference observed between both types of study design were found to be significant (*p* = 0.003) (**Supplementary Material Figure 13**). As for the geographical region, because there were very few studies to allow for a detailed analysis, we obtained variable results, as shown in **Supplementary Material Figure 14**.

## DISCUSSION

The findings of this systematic review and metaanalysis indicate the following:

First, that most authors documented an immediate improvement in oxygenation with prone positioning, albeit with contradictory results regarding whether these changes persisted after resupination.[15;26] A potential risk of this improvement in oxygenation is the undue delay of ETI, which could worsen the patients’ prognosis. In this regard, considering that the findings of the study conducted by Dueñas-Castell et al.[22], indicate that only survivors experienced a significant change in their oxygenation, a lack of this improvement might allow for identifying patients at a high risk of mortality in whom delays in the implementation of ETI or IMV should be avoided. On the other hand, the findings of Kaur et al.’s study[17] showed that this response in patient oxygenation is greater when pronation is applied precociously and Coppo et al.[15] found that time elapsed between hospitalization and pronation was shorter among responders compared with nonresponders. The above suggests that the implementation of prone positioning in the earliest stages of ARDS could improve clinical outcomes.

Second: The pooled ETI rate was 29.3%, with a range of 10.0% to 57.8%, although no statistically significant differences were found according to the geographical region or the study design.

Although we estimated an overall protective effect of prone positioning against the need for ETI (RR = 0.79; 95% CI: 0.631.00), our result was not statistically significant (p = 0.051). The sensitivity analysis performed after excluding Padrao et al.’s study [24] yielded an overall RR of 0.73 (95% CI: 0.590.91), thus confirming the statistically significant effect of pronation. A possible explanation for this effect is that prone positioning improves clinical outcomes when combined with other measures aimed at increasing oxygen transport, such as HFNC or NIMV, which were not used in this study, while over 60% of the pronated patients received respiratory support with a Ventimask (VMK) with a reservoir, for >4 hours in the case of 58% of them. In addition, the population described in this study had a high morbidity (54% of the patients were obese and another 54% suffered from HBP). Conversely, the results reported by PerezNieto et al. [23] regarding a population of patients with less comorbidities (14.4% were obese, 2.1% had a heart disease, and 34.5% suffered from HBP) and treated with pronation for 12 hours showed a lower rate of ETI among the group of pronated patients and allowed to conclude that the independent variables associated with ETI were the patients’ age, a low baseline SpO_2_/FiO_2_, and the use of a VMK with a reservoir (37.6% of the pronated patients in their case series).

An analysis of the differences in the RR according to the study design revealed that the lowest protective effect of pronation was reported by the only prospective observational study included in the analysis,[14] in which case patients with severe hypoxia (PaO_2_/FiO_2_ = 125.0) homogeneously supported with HFNC therapy were pronated for >16 hours per day.

The metaanalysis of the results obtained from the RCTs concluded that pronation did have a statistically significant protective effect against the need for ETI (RR = 0.83; 95% CI: 0.710.97), which reinforces the beneficial impact of this strategy.

Third: The pooled mortality rate was 16.6%, although studies with a smaller sample size tended to report lower rates. However, it should be noted that no significant differences were found in this rate according to the study design or the geographical region, although our analysis included a limited number of studies by region to allow for drawing conclusions.

The overall results indicate a significant protective effect of prone positioning against mortality (RR = 0.69; 95% CI: 0.480.99). Despite the fact that the sensitivity analysis revealed no remarkable changes, PérezNieto et al.’s study[23] was identified as that with the greatest impact on the overall result, as a RR of 0.77 (95% CI: 0.511.18) was observed after excluding it. This corresponded to a retrospective, multicenter study including 827 patients with a mean age of 54.3 years, 505 of whom (baseline SpO_2_/FiO_2_ of 189.5) were pronated for at least 2 hours, in addition to receiving HFNC or NIMV therapy, in which an association between prone positioning and a lower risk of mortality was concluded. The opposite effect was found in Ehrmann et al.’s study [26] a metatrial including a large sample of international patients with a greater mean age (61.1 years) and more severe hypoxia (baseline SpO_2_/FiO_2_ = 148.2) who were treated with HFNC therapy and in whom the implementation of prone positioning for as long as possible each day had a favorable effect on ETI, without increasing the risk of death (hazards ratio [HR] = 0.87; 95% CI: 0.681.11) at 28 days.

The analysis performed according to the study design yielded a RR >1 for the RCTs with small sample sizes and a RR of around 0.5 for the cohort studies, which was deemed to be the global effect; that is, a 50% decrease in patient mortality. The beneficial impact of this strategy identified in observational studies suggests that this procedure might even reduce mortality in standard clinical practice conditions.

As variables related to a greater risk of mortality, Kaur et al. [17] described advanced age, the use of IMV, treatment with hydrocortisone, and a greater time elapsed between the onset of HFNC therapy and the implementation of the pronation maneuver. Dueñas-Castell et al. [22] also described clinical variables associated with greater mortality, including a ROX index ≤4.5, a SOFA score ≥6, and a SpO_2_ ≤89% before pronation, and a respiratory rate ≥24 breaths per minute (bpm) and a SpO_2_ ≤92% after pronation.

Other recent studies have also analyzed the benefits of prone positioning in patients with hypoxia secondary to a SARSCoV2 infection. The metaanalysis conducted by Chua et al. [2], for example, only included observational studies and indicated that prone positioning improved both the PaO_2_/FiO_2_ and SpO_2_ compared with supine positioning in COVID19 patients (whether or not intubated), and that it significantly reduced the mortality rate, but not the incidence of ETI. More recently, when analyzing the subgroup of RCTs, we found that Beran et al. [3] identified a significant effect of pronation in reducing the need for ETI. Our metaanalysis corroborates these findings and our results show that the overall effect of awake prone positioning on the need for ETI in COVID19 patients could be statistically significant when combined with HFNC or NIMV therapy.

It should be noted that this systematic review and metaanalysis has some limitations, including the fact that, although this is a metaanalysis including a large number of patients, over half of the studies were observational and retrospective in nature, and with small sample sizes, albeit with high quality assessment scores. Although there are similar metaanalyses, they only include RCTs; therefore, ours updates their findings. In the reviewed articles, there is a lack of data on the comorbidities, frailty, and clinical phenotype of the patients with respiratory failure secondary to COVID-19 who could respond to the pronation maneuver. Likewise, there is also a lack of data concerning other frequent events, such as bacterial superinfection or pulmonary embolism, which could have an impact on the patients’ clinical evolution. Regarding the incidence of mortality, our results indicate the existence of a publication bias, with a tendency to the publication of studies with a greater sample size and mortality rate. In addition, the studies were not designed to evaluate the effect of the duration of the prone positioning.

Clinical trials with close monitoring of the response parameters to prone positioning and its tolerance in awake patients with hypoxia secondary to COVID-19 are required to allow for defining the benefits linked to HFNC or NIMV support, in addition to the population groups that might benefit from this strategy, thus also avoiding delayed ETI in cases in which this procedure is necessary.

## CONCLUSIONS

Awake prone positioning improved oxygenation in most patients with hypoxia due to COVID19 who were included in the majority of the studies analyzed in this review. However, the persistence of this response following resupination has barely been evaluated and the results reported in this regard are contradictory. This improvement seems to be associated with early application of the maneuver.

A protective effect of prone positioning against the need for IMV was observed, although without statistical significance, with the exception of the subgroup of RCTs. However, the pooled results suggest that patients’ response to awake prone positioning could be greater when this procedure is implemented in a timely manner and in combination with NIMV or HFNC.

Overall, a protective effect of pronation against mortality was estimated. In fact, this protective effect has been proven in observational studies, which suggests that this strategy could have a beneficial impact on mortality in standard clinical practice conditions.
